# Molecular Engineering of Silk via Fatty Acid Esterification for Water-Stable, Anti-fouling, Biocompatible, Implantable Materials

**DOI:** 10.64898/2026.10.20.760528

**Published:** 2026-10-21

**Authors:** Sanjana Gopalakrishnan, Emilee Zhang, Mariah L. Arral, Edward B. Gordon, Sophie M. Steedly, Jugal K. Sahoo, Chunmei Li, David L. Kaplan

## Abstract

Biopolymers offer attractive properties for implantable materials, but their limited aqueous and enzymatic stability constrains long-term performance in biological environments. Silk fibroin is mechanically tunable and thermoplastically processable, yet remains susceptible to swelling in aqueous environments, which can impact mechanical properties. Here, we establish fatty acid esterification as a strategy to transform silk into a hydrophobic, water-stable, yet still bioresorbable material (FAsilk). Fatty acid functionalization significantly increased resistance to aqueous swelling and enzymatic degradation while preserving structural and mechanical integrity and thermoplastic processability. The resulting FAsilk biomaterials also reduced microbial contamination, blood clot formation, and macrophage adhesion relative to native silk, due to the change in hydrophobicity, while maintaining cytocompatibility and hemocompatibility. Subcutaneous implantation in mice demonstrated favorable biological responses, including minimal impact on body weight and no significant pro-inflammatory responses, indicating no adverse effects on animal health. Thus, fatty acid functionalization enables silk to combine hydrophobicity and aqueous stability with processability and biodegradability, providing a mechanically robust materials platform for bioresorbable implants, including medical devices, tissue-engineered grafts and drug delivery systems.

